# Coming down from the trees: Is terrestrial activity in Bornean orangutans natural or disturbance driven?

**DOI:** 10.1038/srep04024

**Published:** 2014-02-13

**Authors:** Marc Ancrenaz, Rahel Sollmann, Erik Meijaard, Andrew J. Hearn, Joanna Ross, Hiromitsu Samejima, Brent Loken, Susan M. Cheyne, Danica J. Stark, Penny C. Gardner, Benoit Goossens, Azlan Mohamed, Torsten Bohm, Ikki Matsuda, Miyabi Nakabayasi, Shan Khee Lee, Henry Bernard, Jedediah Brodie, Serge Wich, Gabriella Fredriksson, Goro Hanya, Mark E. Harrison, Tomoko Kanamori, Petra Kretzschmar, David W. Macdonald, Peter Riger, Stephanie Spehar, Laurentius N. Ambu, Andreas Wilting

**Affiliations:** 1 HUTAN/Kinabatangan Orangutan Conservation Programme, PO Box 17793, 88874 Kota Kinabalu, Sabah, Malaysia; 2 Sabah Wildlife Department, Wisma Muis, 88100 Kota Kinabalu, Sabah, Malaysia; 3 Borneo Futures Project, People and Nature Consulting International, Ciputat, Jakarta, 15412, Indonesia; 4 North England Zoological Society, Chester Zoo, Chester, UK; 5 North Carolina State University, Department of Forestry and Environmental Resources, Turner House, Campus Box 7646, Raleigh, NC 27695, USA; 6 Leibniz Institute for Zoo and Wildlife Research, Alfred-Kowalke-Straβe 17, 10315 Berlin, Germany; 7 School for Archaeology and Anthropology, Building 014, Australian National University, Canberra, ACT 0200, Australia; 8 Center for International Forestry Research, P.O. Box 0113 BOCBD, Bogor 16000, Indonesia; 9 Wildlife Conservation Research Unit, Department of Zoology, University of Oxford, The Recanati-Kaplan Centre, Tubney House, Abingdon Road, Tubney, Abingdon, OX13 5QL, UK; 10 Center for Southeast Asian Studies, Kyoto University, Kyoto, Japan; 11 School of Resource and Environmental Management, Simon Fraser University, Burnaby, British Columbia, Canada; 12 Integrated Conservation, Gig Harbor, Washington; 13 Orangutan Tropical Peatland Project, Jl. Semeru 91, Palangka Raya 73112, Central Kalimantan, Indonesia; 14 Danau Girang Field Centre, c/o Sabah Wildlife Department, Wisma Muis, 88100 Kota Kinabalu, Sabah, Malaysia; 15 Organisms and Environment Division, School of Biosciences, Cardiff University, Sir Martin Evans Building, Museum Avenue, Cardiff CF10 3AX, UK; 16 WWF–Malaysia, 49, Jalan SS23/15, 47400 Petaling Jaya, Selangor, Malaysia; 17 Primate Research Institute, Kyoto University Inuyama, Aichi 484-8506, JAPAN; 18 Wildlife Research Center of Kyoto University, 3rd Floor, 2-24 Tanaka-Sekiden-cho, Sakyo, Kyoto, 606-8203, Japan; 19 WWF-Malaysia, CPS Tower, Centre Point Complex, 88000 Kota Kinabalu, Sabah, Malaysia; 20 Unit for Primate Studies-Borneo, Institute for Tropical Biology and Conservation, Universiti Malaysia Sabah, Jalan UMS, 88400 Kota Kinabalu, Sabah, Malaysia; 21 Biodiversity Research Centre; University of British Columbia; Vancouver, BC, Canada; 22 Research Centre in Evolutionary Anthropology and Palaeoecology, School of Natural Sciences and Psychology, Liverpool John Moores University, Liverpool, UK; 23 Sumatran Orangutan Conservation Programme, PanEco/YEL, Jl. Wahid Hasyim 51/74, 20154 Medan, North Sumatra, Indonesia; 24 Department of Geography, University of Leicester, University Road, Leicester, LE1 7RH, UK; 25 Houston Zoo, Texas, USA; 26 Anthropology Program, University of Wisconsin Oshkosh, Oshkosh, WI, 54901, USA; 27 These authors contributed equally to this work.

## Abstract

The orangutan is the world's largest arboreal mammal, and images of the red ape moving through the tropical forest canopy symbolise its typical arboreal behaviour. Records of terrestrial behaviour are scarce and often associated with habitat disturbance. We conducted a large-scale species-level analysis of ground-based camera-trapping data to evaluate the extent to which Bornean orangutans *Pongo pygmaeus* come down from the trees to travel terrestrially, and whether they are indeed forced to the ground primarily by anthropogenic forest disturbances. Although the degree of forest disturbance and canopy gap size influenced terrestriality, orangutans were recorded on the ground as frequently in heavily degraded habitats as in primary forests. Furthermore, all age-sex classes were recorded on the ground (flanged males more often). This suggests that terrestrial locomotion is part of the Bornean orangutan's natural behavioural repertoire to a much greater extent than previously thought, and is only modified by habitat disturbance. The capacity of orangutans to come down from the trees may increase their ability to cope with at least smaller-scale forest fragmentation, and to cross moderately open spaces in mosaic landscapes, although the extent of this versatility remains to be investigated.

The Bornean orangutan *Pongo pygmaeus* is the largest arboreal species in the world and its survival is linked to forest habitat^1^,^2^. Despite the orangutan's iconic value and millions of dollars spent annually on its conservation^3^, the species is declining throughout its range. In Borneo, more than 70% of orangutans occur in fragmented multiple-use and human-modified forests that have lost many of their original ecological characteristics^4^,^5^. The consequences of these drastic habitat changes on orangutan survival, behaviour and ecology are only just starting to be documented^3^,^4^,^6^,^7^. Some authors have proposed that forest degradation may force the species to the ground more frequently^8^,^9^. We can suppose that increased terrestriality would increase predation risk, interactions with and persecution by humans, and exposure to novel pathogens. On the other hand, terrestrial behaviour could also facilitate movement and, therefore, dispersal, especially in degraded or fragmented landscapes as a result of natural or man-made processes. It could also create new opportunities to access different food sources^10^. Ultimately, a better understanding of the drivers of orangutan terrestriality and how this influences dispersal and movement is important for designing effective landscape management strategies for maintaining viable meta-populations of this species in Borneo^4^.

Most studies of orangutan locomotion have been based on direct observations^11^,^12^. However, orangutans may be reluctant to come to the ground in the presence of human observers, and remote camera traps present an opportunity to overcome this potential bias^8^. We conducted a large-scale species-level analysis of ground-based camera-trapping data to evaluate the extent to which Bornean orangutans travel terrestrially and to investigate possible drivers for this behaviour.

## Results

Altogether we collected camera-trapping data from 16 study areas from Sabah (Malaysia) and East and Central Kalimantan (both Indonesia), for which reliable orangutan density estimates were available (Table 1). Pictures were collected between June 2006 and March 2013, and included data from all months of the year. The total dataset encompassed 159,152 trap days at 1,409 independent camera-trap stations.

**Figure 1:**
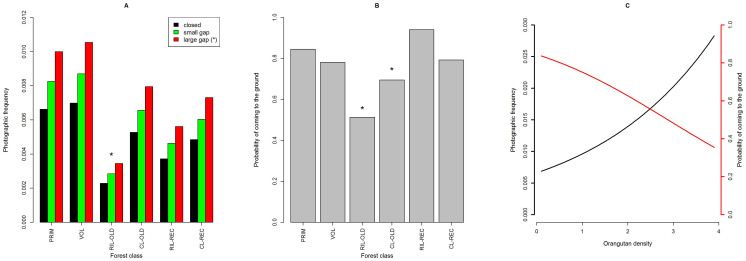
(A) Photographic frequency (including different camera-trap placement categories) and (B) probability of orangutans coming to the ground for six different forest classes ordered from primary to recently heavily logged forest (PRIM = primary forest; VOL = very old conventionally logged forest >20 years ago; RIL-OLD = reduced impact logging 2–20 years ago; CL-old = conventional logging 2–20 years ago; RIL-REC = reduced impact logging within the last 2 years; RIL-REC = conventional logging in the last 2 years). (C) Relationship of photographic frequency (black) and probability of orangutans coming to the ground (red) with orangutan density, plotted for primary forest, but patterns for other forest classes are equivalent.

Orangutans were recorded on the ground in all forest classes, indicating that terrestrial activity occurs regardless of habitat disturbance. Nevertheless, the regression model revealed that forest class, camera-trap placement and orangutan density influenced the photographic frequency and the probability of orangutans coming to the ground (Table 3, Figure 1 A–C). Photographic frequencies were significantly higher beneath large canopy gaps than under closed canopy (Figure 1A & 1B, Table 3). In 428 out of 641 orangutan records the sex-age class could be reliably determined (see Methods). We observed females alone 27 times, females with clinging babies or with walking young 63 and 25 times, respectively, unflanged males 48 times, and flanged males 265 times. We recorded flanged males significantly more often than expected based on their proportion in the population (see Methods) (χ^2^ = 32.050, df = 1, p < 0.001), suggesting they are more terrestrial than unflanged males, and females. Only 15 orangutan records were obtained during night time (before 0600 h and after 1800 h).

## Discussion

Overall, Bornean orangutan terrestrial activity appears more common than previous anecdotal observations suggested^8^, which indicates that the species exhibits a flexible and varied repertoire of locomotion. This is supported evolutionarily: the potential ancestors and fossil relative taxa of *Pongo*, are thought to have used more ground locomotion than the current *Pongo*^14^,^15^. This, in turn, has led to the suggestion that ancestral orangutans may have been able to cover larger distances on the ground^13^,^16^. It should be noted that the data used in the present analysis come predominantly from the subspecies *P. pygmaeus morio* in northeastern Borneo and there is the potential for regional differences in the species' response to forest characteristics driven by differences in ecological circumstances^13^. For example, an analysis of raw model residuals by study site (Supplementary Information) showed stronger negative outliers for photographic counts from the Sabangau peat swamp (i.e., subspecies *P. p. wurmbi*) than from other sites, suggesting that swamp habitat may reduce terrestriality in orangutans. Further, preliminary data indicate that the Sumatran orangutan *Pongo abelii* is much less terrestrial, possibly because of the presence of tigers *Panthera tigris* as potential predators, which are absent from Borneo^1^.

Our analysis shows that the degree of terrestriality is modulated by forest structure. This suggests that anthropogenic canopy disruptions will increase terrestrial activity in orangutans, but habitat disturbance is not the only driver for this behaviour. Indeed, both photographic frequencies and probabilities of coming to the ground were lowest in areas logged by reduced impact logging schemes between two and 20 years ago and not, as expected if terrestriality were determined by disturbance, in primary forests. This may be caused by a continuous lower canopy layer that develops after reduced impact logging and is relatively easy to travel through without coming to the ground to cross gaps. In contrast, the irregular canopy structure in primary forests and the large man-made gaps in forests recently logged using conventional logging methods may increase the energetic costs of arboreal locomotion^11^, offering an explanation for similar terrestriality in these forest classes. Although our data also support the perception that terrestriality in orangutans is most prevalent in larger and heavier flanged males^9^, our camera trap data showed that all age-sex classes travel on the ground irrespective of habitat type.

The ability to cross forest gaps, especially for males, which are the dispersing sex^17^,^18^,^19^, suggests that gene flow can occur even in disturbed and possibly fragmented habitats^10^. In addition to occasional advantages in travelling from place to place, terrestriality could enhance the possibilities for foraging for terrestrial resources, such as succulent shoots, termites or mineral clay^10^,^20^. This may be particularly important during periods of habitat-wide fruit scarcity. Heightened orangutan terrestriality in human-modified landscapes and the resulting increase in contact with people creates new risks, such as increased susceptibility to hunting and exposure to new diseases, as great apes are vulnerable to many human diseases^21^,^22^.

Our findings reinforce the importance of incorporating degraded forests recovering from logging disturbance into orangutan conservation strategies^3^,^4^,^6^. The capacity of orangutans to come down from the trees may increase their ability to cope with at least smaller scale fragmentation^10^, and to cross moderately open spaces in mosaic landscapes, although the extent of this versatility remains to be investigated. In order to design conservation management strategies that will allow for the species to persist in anthropogenic landscapes, however, practitioners need to be mindful of the potential risks associated with terrestrial activities in orangutans. The “man of the forest” cannot be regarded as safely tucked away up in the trees.

## Methods

Our analysis is based on a compilation of existing ground-based camera trapping data that had been collected at 16 sites in Borneo, mostly on the northern part of the island (Table 1).

### Data base

To avoid inflated counts caused by repeated detections of the same individual, only one record per hour per camera site was included in the data analysis. We excluded all cameras that were placed at salt licks, and data from the orangutan rehabilitation site at Sepilok Forest Reserve, as we assumed that both factors could influence terrestrial behaviour (i.e., a particular resource for the former^20^ and habituation to humans for the latter^23^). To avoid spatial autocorrelation in the data, we only included camera-trap stations that were a minimum of 1 km apart from each other, assuming that orangutans do not move continuously along the ground over distances in the order of a kilometre^10^, so that events to come to the ground are independent at that scale. The independence of these observations would be compromised if orangutans routinely moved along the ground over 1 km. This assumption is backed by the observation that the *overall* (i.e. arboreal and terrestrial) maximum distance moved by orangutans within a day is in most cases below 1 km^24^. Considering these restrictions all analyses are based on 641 independent orangutan records taken at 1,409 stations during 159,152 trap days (see Table 1).

### Analysis

All analyses were performed in R, version 2.15.1^25^.

#### Terrestrial activity as a function of forest disturbance and density

We hypothesised that regional forest structure and camera-trap site specific canopy structure (also referred to as camera-trap placement), as well as orangutan density, could influence how often orangutans are photographed on the ground. We therefore classified forests into six classes, based on their current and past management history: 1. primary forest (PRIM; not disturbed); 2. very old conventionally logged forest (VOL; last logging more than 20 years ago); 3. old slightly logged forest (OLD-RIL; exploitation using reduced impact logging (RIL) practices between 2–20 years ago); 4. old heavily logged forest (OLD-CL; conventional logging practices 2–20 years ago); 5. recent slightly logged forest (REC-RIL; RIL within the last 2 years); 6. recent heavily logged forest (REC-CL; conventional logging in the last 2 years). We further categorized camera trap placement according to Loken *et al.*^8^ as under closed canopy (0–3 m gaps that orangutans are likely able to cross by tree swaying), small canopy gap (3–5 m gaps that orang-utans might be able to cross by tree swaying), or large canopy gap (>5 m gaps that orang-utans are unlikely to cross by tree swaying). Gap size was determined post hoc based on field notes and photographs. We recognize that there might be some inherent error in classifying gap size post hoc, but the coarse classification of gap size should buffer most of that error. Orangutan densities were obtained from the literature and from unpublished data of the authors for each site (Table 1). We acknowledge that density estimates were obtained with different methods and that some of these methods are controversial; however, the estimates used here represent the best currently available data for these sites and are widely used to assess the status of the species.

To quantify the influence of the above variables on orangutan terrestriality, we compiled the number of orangutan records taken at each camera location and analysed the data using a zero-inflated Poisson (ZIP) regression^26^. A ZIP model allows for overdispersion in counts in the form of excess zeros, which we observed in our data set. It is a Binomial-Poisson mixture that attempts to separate zero counts into structural zeros (sites where orangutans never come to the ground so that we can only observe a zero count) and sampling zeros (sites where orangutans do come to the ground but we happen to not record them there). The binomial component of the ZIP model estimates the ***probability of coming to the ground*** at a given camera trap station. The Poisson component of the ZIP model describes the number of records we expect to observe (referred to as the ***photographic frequency***) at a camera trap, conditional on the species coming to the ground at all. Both parameters can be modelled as functions of covariates on the logit and log scale, respectively. We used station-specific survey effort (i.e., camera-trap days) as offset, and orangutan density, forest class, and camera-trap placement (characterised by station-specific canopy gap size) as model covariates.

We built a number of models, differing in the combination of explanatory covariates (forest class, canopy gap size and orangutan density), and used the Akaike Information Criterion (AIC) to select the most parsimonious (‘best’) model. Because we expect a direct relationship between the sampling effort at a camera-trap station and the number of pictures obtained, all models used the number of camera trap days each station was surveyed as an offset in the model for frequency.

To reduce the total number of candidate models, we first explored different models for the photographic frequency while holding the probability of coming to the ground constant (i.e. no explanatory variables of the probability of coming to the ground). Conditional on the best frequency model we then built candidate models for the probability of coming to the ground (Table 2). The best model for photographic frequency contained all three covariates. Conditional on this frequency model, the best overall model additionally contained effects of forest class and density on the probability of coming to the ground. The second-best model, which also included camera placement as a covariate on the probability of coming to the ground, only had a delta AIC relative to the best model of 0.66 and was therefore essentially equally supported by the data (Table 2). Since we were unable to determine a single best model, we employed model averaging, where parameter estimates are obtained as a weighted average over all candidate models^27^.

#### Age-sex classes on the ground

To investigate whether terrestrial behaviour is exhibited by all demographic classes (i.e. flanged males, unflanged males, females and females with offspring) we would have ideally run separate ZIP regressions for different classes, but identification of these classes was only possible with high certainty in about 50% of all camera trap records. We cannot assume that failure to identify the age-sex class occurs at random – it is much harder to distinguish a small male from a female, or ascertain that a female is with offspring, than it is to unambiguously identify a flanged male. The only analysis we conducted with respect to demographic class was, therefore, a comparison of the observed versus expected number of flanged male pictures using a Chi-square test. We expect a 50:50 male-female ratio in the orangutan population^28^; within the males, on Borneo there are typically 1.6:1 flanged to unflanged males^28^,^29^. Thus, if all demographic groups came to the ground relative to their occurrence in the population, we would expect flanged male pictures to comprise 31% of our total sample. We considered all pictures in which the demographic group could not be identified as “not flanged male” – this is unlikely to be true since pictures that only show small part of an orangutan could be of a flanged male. However, this procedure guaranteed a conservative approach towards the question of whether flanged males come to the ground more frequently than expected.

## Author Contributions

M.A., R.S., E.M. and A.W. wrote the main manuscript text, R.S. and A.W. performed the statistical analysis and R.S. prepared the figures. A.J.H., J.R., H.S., B.L., S.M.C., D.J.S., B.G., P.C.G., A.M., T.B., I.M., M.N., S.K.L., H.B., J.B., S.W., G.F., G.H., M.E.H., T.K., P.K. and S.S. contributed data. B.G., D.W.M., P.R. and L.N.A. facilitated the fieldwork. All authors contributed to the development of the manuscript, and all authors reviewed it.

## Supplementary Material

Supplementary Information Supplementary Methods

## Figures and Tables

**Table 1:** Summary statistics for orangutan camera trapping data from Borneo used in the present analysis. For definition of forest classes, see Methods

No	Study site	Status^1^	State	No of stations	No of trap days	No of records	Forest class	Orangutan density [ind/km^2^]	Density reference
1	Bawan Forest	CFR	Central Kalimantan, Indonesia	65	2,064	2	REC-RIL	2.15	30
2	Croker Range Park	NP	Sabah, Malaysia	35	3,999	0	PRIM & VOL	1.0	5
3	Danum Valley Conservation Area	TPA	Sabah, Malaysia	198	20,223	51	PRIM	1.0	5
4	Deramakot Forest Reserve	CFR	Sabah, Malaysia	144	10,532	25	VOL & OLD-RIL	1.5	5
5	Lower Kinabatangan Wildlife Sanctuary	WS	Sabah, Malaysia	128	19,602	179	VOL	1.1–3.9^2^	31
6	Kuamut Forest Reserve	CFR	Sabah, Malaysia	38	1,949	2	REC-CL	0.1–1.4^2^	5
7	Kutai National Park	NP	East Kalimantan Indonesia	53	3,310	42	VOL	1.0–1.3^2^	Spehar, pers. com.
8	Maliau Basin	TPA	Sabah, Malaysia	27	5,232	0	PRIM & OLD-REC	0.1	5
9	Malua Forest Reserve	CFR	Sabah, Malaysia	107	9,730	40	REC-CL	1.3–1.6^2^	5
10	Sabangau Peat Swamp Forest	NP	Central Kalimantan, Indonesia	58	26,722	49	OLD-RIL	1.7	32
11	Segaliud Lokan Forest Reserve	CFR	Sabah, Malaysia	67	3,452	19	OLD-CL & REC-RIL	1.2	5
12	Kulamba Wildlife Reserve	WR	Sabah, Malaysia	4	252	2	VOL	2.3	5
13	Tabin Wildlife Reserve	WR	Sabah, Malaysia	283	28,462	104	VOL	1.3	5
14	Tangkulap Forest Reserve	CFR	Sabah, Malaysia	100	6,057	37	OLD-CL	0.6	5
15	Ulu Segama Forest Reserve	CFR	Sabah, Malaysia	61	9,829	13	OLD-CL	1.1–1.4^2^	6
16	Wehea Forest	CFR	East Kalimantan, Indonesia	41	7,737	76	OLD-RIL	1.1	Loken, pers. com.
	TOTAL			1,409	159,152	641			

^1^CFR = Commercial Forest Reserve; NP = National Park; TPA = Totally Protected Area; WS = Wildlife Sanctuary; WR = Wildlife Reserve.

^2^Density varied between areas.

**Table 2:** AIC summaries for zero-inflated Poisson regression of photo-counts of orangutans; models for the probability of coming to the ground as a function of forest class (*for*), camera trap placement (*cam*) and orangutan density (*dens*), or no covariates (0), conditional on best photographic frequency model containing all covariates. For definition of forest classes and camera trap placement, see Methods

Model	No. parameters	AIC	delta AIC	AIC weight
*for + dens*	16	2550.120	0.000	0.533
*for + cam + dens*	18	2550.783	0.664	0.364
*cam + dens*	13	2554.802	4.683	0.055
*For*	15	2555.798	5.678	0.032
*for + cam*	17	2557.085	6.965	0.016
*Dens*	11	2565.759	15.640	0.000
*Cam*	12	2567.900	17.781	0.000
0	10	2573.684	23.565	0.000

**Table 3:** Model-averaged parameter estimates from zero-inflated Poisson regression of photographic frequencies of orangutans against forest class (PRIM, VOL, RIL-OLD, CL-OLD, RIL-REC, CL-REC, see Methods for abbreviations), camera trap placement (closed canopy, small gap, large gap) and orangutan density. Primary forest and closed canopy were reference categories in the regression

Parameter	Coefficients	Estimate	SE	Lower CI	Upper CI	z value	p value
Frequency (log scale)	Intercept	−5.017	0.227	−5.462	−4.572	22.098	<0.001
	*β* (VOL)	0.053	0.242	−0.423	0.528	0.217	0.828
	*β* (RIL-OLD)	−1.064	0.300	−1.653	−0.475	3.542	<0.001
	*β* (CL-OLD)	−0.229	0.302	−0.821	0.363	0.758	0.448
	*β* (RIL-REC)	−0.578	1.165	−2.862	1.706	0.496	0.620
	*β* (CL-REC)	−0.313	0.332	−0.963	0.337	0.943	0.346
	*β* (small gap)	0.220	0.152	−0.079	0.518	1.443	0.149
	*β* (large gap)	0.412	0.179	0.061	0.762	2.300	0.021
	*β* (density)	0.372	0.106	0.166	0.579	3.529	<0.001
Probability (logit scale)	Intercept	1.694	0.337	1.033	2.355	5.022	<0.001
	*β* (VOL)	−0.423	0.330	−1.070	0.224	1.283	0.200
	*β* (RIL-OLD)	−1.643	0.456	−2.537	−0.749	3.603	<0.001
	*β* (CL-OLD)	−0.871	0.380	−1.615	−0.127	2.294	0.022
	*β* (RIL-REC)	1.070	1.420	−1.714	3.854	0.754	0.451
	*β* (CL-REC)	−0.353	0.456	−1.247	0.542	0.773	0.439
	*β* (small gap)	−0.461	0.300	−1.050	0.127	1.538	0.124
	*β* (large gap)	−0.025	0.398	−0.805	0.756	0.062	0.951
	*β* (density)	−0.587	0.217	−1.013	−0.162	2.706	0.007

## References

[b1] Delgado R. A. & van Schaik C. P. The behavioral ecology and conservation of the orangutan (*Pongo pygmaeus*): a tale of two islands. Evol. Anthropol. 9, 201–218 (2000).

[b2] MacKinnon J. R. The behavior and ecology of wild orangutans (*Pongo pygmaeus*). Anim. Behav. 22, 3–74 (1974).

[b3] Meijaard E., Wich S., Ancrenaz M. & Marshall A. J. Not by science alone: why orangutan conservationists must think outside the box. Ann. NY Acad. Sci. 1249, 29–44 (2011). 22175247 10.1111/j.1749-6632.2011.06288.x

[b4] Wich S. A. *et al.* Understanding the impacts of land-use policies on a threatened species: Is there a future for the Bornean orangutan? PLOS ONE 7, e49142 (2012). 23145100 10.1371/journal.pone.0049142 PMC3492325

[b5] Ancrenaz M. *et al.* Aerial surveys give new estimates for orangutans in Sabah, Malaysia. PLOS Biology 3, e3 (2005). 15630475 10.1371/journal.pbio.0030003 PMC534813

[b6] Ancrenaz M. *et al.* Recent surveys in the forests of Ulu Segama Malua, Sabah, Malaysia, show that orangutans (*P. p. morio*) can be maintained in slightly logged forests. PLOS ONE 5, e11510 (2010). 20634974 10.1371/journal.pone.0011510 PMC2901384

[b7] Meijaard E. *et al.* Unexpected ecological resilience in Bornean orangutan and implications for pulp and paper Plantation Management. PLOS ONE 5, e12813 (2010). 20877646 10.1371/journal.pone.0012813 PMC2943906

[b8] Loken B., Spehar S. & Rayadin Y. Terrestriality in the Bornean orangutan (*Pongo pygmaeus morio*) and implications for their ecology and conservation. Am. J. Primatol. 75, 1129–1138 (2013). 23784888 10.1002/ajp.22174

[b9] Rijksen H. D. & Meijaard E. Our Vanishing Relative (Kluwer Academic, 1999).

[b10] Ancrenaz M. *et al.* Of *Pongo*, palms, and perceptions – A multidisciplinary assessment of orangutans in an oil palm context. Oryx (in press).

[b11] Thorpe S. K. S., Crompton R. H. & Alexander R. M. Orangutans use compliant branches to lower the energetic cost of locomotion. Biol. Lett. 3, 253–256 (2007). 17439848 10.1098/rsbl.2007.0049 PMC2464692

[b12] Thorpe S. K. S. & Crompton R. H. [Orang-utan positional behaviour]. Orangutans: Geographic Variation in Behavioural Ecology and Conservation [Wich, S. A., Utami, S. S., Mitra Setia, T. & van Schaik, C. P. (eds.)] [33–46] (Oxford University Press, Oxford, 2009).

[b13] Wich S. A., Utami S. S., Mitra Setia T. & van Schaik C. P. Orangutans: Geographic Variation in Behavioural Ecology and Conservation (Oxford University Press, Oxford, 2009).

[b14] Begun D. R. & Kivell T. L. Knuckle-walking in *Sivapithecus*: the combined effects of homology and homoplasy and implications for the origin of human bipedalism. J. Hum. Evol. 60,158–170 (2011). 21185062 10.1016/j.jhevol.2010.10.002

[b15] Harrisson M. E. & Chivers D. J. The orang-utan mating system and the unflanged male: a product of increased food stress during the late Miocene and Pliocene? J. Hum. Evol. 52, 275–293 (2006). 17083968 10.1016/j.jhevol.2006.09.005

[b16] Von Koenigswald G. H. R. [Distribution and evolution of the orangutan, *Pongo pygmaeus*]. The Orangutan: Its Biology and Conservation, [de Boer, L. E. M. (ed.)] [1–15] (Dr W. Junk Publishers, the Hague, 1982).

[b17] Nater A. *et al.* Sex-biased dispersal and volcanic activities shaped phylogeographic patterns of extant orangutans (genus: *Pongo*). Mol. Biol. Evol. 28, 2275–2288 (2011). 21335339 10.1093/molbev/msr042

[b18] Arora N. *et al.* Parentage-based pedigree reconstruction reveals female matrilineal clusters and male-biased dispersal in nongregarious Asian great apes, the Bornean orang-utans (*Pongo pygmaeus*). Mol. Ecol. 21, 3352–3362 (2012). 22647036 10.1111/j.1365-294X.2012.05608.x

[b19] Nietlisbach P. *et al.* Heavily male-biased long-distance dispersal of orang-utans (genus: *Pongo*), as revealed by Y-chromosomal and mitochondrial genetic markers. Mol. Ecol. 21, 3173–3186 (2012). 22463133 10.1111/j.1365-294X.2012.05539.x

[b20] Matsubayashi H. *et al.* Natural-lick use by orangutans and conservation of their habitats in Bornean tropical production forests. Raffles Bull. Zool. 59, 109–115 (2011).

[b21] Muehlenbein M. P. & Ancrenaz M. Minimizing pathogen transmission at primate ecotourism destinations: the need for input from travel medecine. J. Trav. Medic. 16, 229–232 (2009). 10.1111/j.1708-8305.2009.00346.x 19674260

[b22] Ryan S. J. & Walsh P. D. Consequences of non-intervention for infectious disease in African great apes. PLOS ONE 6, e29030 (2011). 22216162 10.1371/journal.pone.0029030 PMC3245243

[b23] Russon A. E. [Orangutan rehabilitation and reintroduction]. Orangutans: Geographic Variation in Behavioral Ecology and Conservation [Wich, S., Utami, S., Setia, T. & van Schaik, C. (eds.)] [327–350] (Oxford University Press, Oxford, 2009).

[b24] Singleton I., Knott C. D., Morrogh-Bernard H. C., Wich S. A. & van Schaik C. P. [Ranging behavior of orangutan females and social organization]. Orangutans: Geographic Variation in Behavioral Ecology and Conservation [Wich, S., Utami, S., Setia, T. & van Schaik, C. (eds.)] [205–212] (Oxford University Press, Oxford, 2009).

[b25] R Core Team R: A language and environment for statistical computing. R Foundation for Statistical Computing, Vienna, Austria.ISBN 3-900051-07-0, URL http://www.R-project.org/ (2012).

[b26] Gelman A. & Hill J. Data Analysis Using Regression and Multilevel/Hierarchical Models, First Edition (Cambridge University Press, Cambridge, 2006).

[b27] Burnham K. P. & Anderson D. R. Model Selection and Multimodel Inference: A Practical Information-Theoretic Approach (2nd ed.) (Springer-Verlag, 2002).

[b28] Marshall A. J. *et al.* [Orangutan population biology, life history, and conservation]. Orangutans: Geographic Variation in Behavioral Ecology and Conservation [Wich, S., Utami, S., Setia, T. & van Schaik, C. (eds.)] [311–326] (Oxford University Press, Oxford, 2009).

[b29] Bruford M. W. *et al.* Projecting genetic diversity and population viability for the fragmented orang-utan population in the Kinabatangan floodplain, Sabah, Malaysia. End. Spec. Res. 12, 249–261 (2010).

[b30] Harrison M. E. *et al.* Preliminary Assessment of the Biodiversity and Conservation Value of the Bawan Forest, Central Kalimantan, Indonesia. Orangutan Tropical Peatland Project Report. Palangka Raya, Indonesia (2012).

[b31] Ancrenaz M., Goossens B., Gimenez O., Sawang A. & Lackman-Ancrenaz I. Determination of ape distribution and population size with ground and aerial surveys: a case study with orangutans in lower Kinabatangan, Sabah, Malaysia. Anim. Conserv. 7, 375–385 (2004).

[b32] Ancrenaz M. Orangutan aerial survey in Sebangau National Park. WWF report BKSDA/BTNS, Kalimantan (2007).

