# Supplementary material for: Coming down from the trees: Is terrestrial activity in Bornean orangutans natural or disturbance driven?
Source: Sci Rep. 2014 Feb 13;4:4024. doi: 10.1038/srep04024 (PMC3923384; doi:10.1038/srep04024)
Supplement: Supplementary Information — Supplementary Methods [file srep04024-s1.doc]

**Supplementary information**

**Title**

Coming down from the trees:

Is terrestrial activity in Bornean orangutans natural or disturbance driven?

**Authors**

Marc Ancrenaz, Rahel Sollmann, Erik Meijaard, Andrew J. Hearn, Joanna Ross, Hiromitsu Samejima, Brent Loken, Susan M. Cheyne, Danica J. Stark, Penny C. Gardner, Benoit Goossens, Azlan Mohamed, Torsten Bohm, Ikki Matsuda, Miyabi Nakabayasi, Shan Khee Lee, Henry Bernard, Jedediah Brodie, Serge Wich, Gabriella Fredriksson, Goro Hanya, Mark E. Harrison, Tomoko Kanamori, Petra Kretzschmar, David W. Macdonald, Peter Riger, Stephanie Spehar, Laurentius N. Ambu, Andreas Wilting

Supplementary Methods

To investigate the general model fit, as well as possible regional differences in the response of orangutans terrestriality to forest disturbance, we calculated the raw model residuals as described by Moore and Swihart (2005) for occupancy models: we used the probability of coming to the ground times the expected photographic frequency based on model-averaged parameter estimates as expected count for a given camera, and subtracted the expected from the observed photographic frequencies. Residuals near 0 indicate a close fit of the model predictions to the observed data. We plotted raw residuals by study area to explore potential outliers. The grouping of residuals by study site allowed us to identify those sites with the most or strongest outliers and to build hypotheses about which regional factors not considered in our broader scale analysis may have led to these outliers. We plotted the raw residuals as violin plots in R using the package vioplot [S1]. A violin plots combines a box plots with a median marker and box delimiting the interquartile range, with a Kernel density plot showing the probability density of the data at different values. As the raw model residuals for most study sites, based on visual assessment, were very similar and centred on 0, we provide only exemplary plots of three study sites in Figure S1: 1. Ulu Segama as a typical example for most study sites, 2. Sabangau Peat Swamp forest, as this area showed the strongest negative outliers for raw residuals, implying that observed photographic counts were mostly lower than predicted by the model, and 3. Wehea Forest because of a recent study on orangutan terrestriality from that area [S2], and as an example for a site with positive outliers, indicating higher photographic counts than predicted by the model.

Supplementary references

1. Adler, D. (2005). vioplot: Violin plot. R package version 0.2. http://cran.r-project.org/web/packages/vioplot/index.html.

2. Loken, B., Spehar, S. and, Rayadin, Y. (2013). Terrestriality in the Bornean orangutan (*Pongo pygmaeus morio*) and implications for their ecology and conservation. Am. J. Primatol. *75*, 1129-1138.


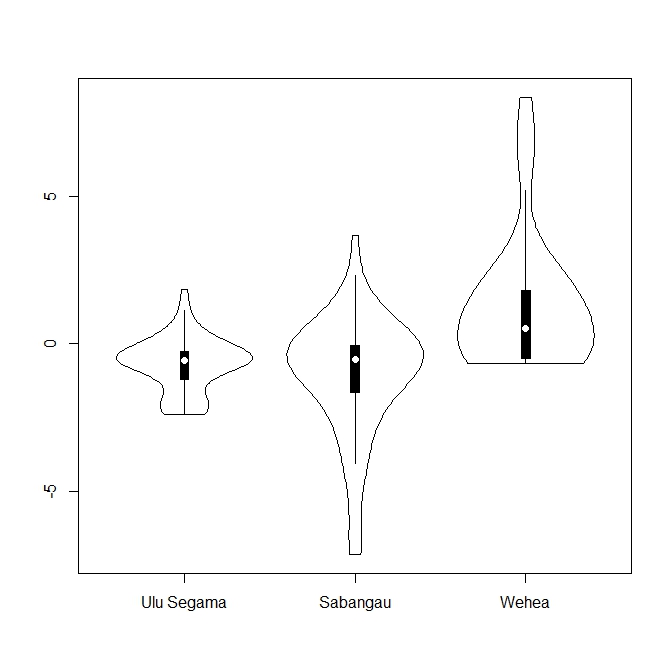
Supplementary figures

Figure S1: Raw model residuals of orangutan photographic counts plotted as violin plots for three study sites from Borneo (Ulu Segama Forest Reserve, Sabangau Peat Swamp Forest and Wehea Forest).
